# Stability of transverse dental arch dimension with passive self-ligating brackets: a 6-year follow-up study

**DOI:** 10.1186/s40510-022-00414-7

**Published:** 2022-06-20

**Authors:** Franz Josef Willeit, Francesca Cremonini, Paul Willeit, Fabio Ramina, Marta Cappelletti, Giorgio Alfredo Spedicato, Luca Lombardo

**Affiliations:** 1 Brunico, Italy; 2 Postgraduate School of Orthodontics, University of Ferrara, via Luigi Borsari, 46, 44121 Ferrara, Italy; 3 Department of Statistics and Quantitative Methods, University of Milano-Bicocca, Milan, Italy

## Abstract

**Objective:**

The stability of the transverse expansion in passive self-ligating bracket treatments is a debated topic in orthodontics. However, to date, only 3 reports are available in the literature, with the maximum follow-up of 3 years after the end of therapy. The present study aims to evaluate the stability of orthodontic treatment with self-ligating brackets in a 6-year follow-up period of time.

**Materials and methods:**

A sample of 56 non-extractive cases (of whom 33 females, mean age 16.9, SD = 9.0 years) consecutively treated with Damon® system was retrospectively selected. All patients received fixed retainers from canine to canine in both arches at the end of treatment, and no removable retainers were provided. The mean values of the transverse intercusp, transverse centroid and transverse lingual distances were evaluated for all teeth from canines to second molars in both arches. Each measure was calculated at four timepoints: before treatment (T0), at the end of treatment (T1), one year after treatment (T2) and six years after treatment (T3). Transverse diameters were measured for all teeth, starting from the canines to the second molars, for a total of 1680 observations, and subsequently compared in order to evaluate intra-treatment and post-treatment modifications.

**Results:**

There were increases in all transverse dental measurements during active treatment. A statistically significant (*p* < .05) reduction of the transverse diameter was found, for upper and lower premolars, from T1 to T3.

**Conclusion:**

The 6-year follow-up analysis detected that the initial transverse expansion showed a statistically significant relapse in premolars. No relapse was detected at the level of canines, due to the presence of fixed retainers, and minimal at first molars.

## Introduction

In order to achieve alignment and leveling, especially in crowded cases, it is necessary to obtain space in dental arches. This space can be obtained by means of orthodontic treatment alternatives including bone-borne-based expansion protocols, the reduction of dental tissue, i.e., the extraction of permanent teeth or interproximal enamel reduction (IPR) [1], elongation of the arch via transverse dental expansion and proclination of the incisors [2, 3].

In fact, the latter option includes treatments performed with self-ligating brackets (SLB), whose ability to expand the arches and the consequent method of correcting malocclusions has generated numerous debates in recent years [4–6].

One recent systematic review compared SLB to conventional brackets (CB) regarding their effectiveness on transversal changes. Meta-analyses found out that SLBs were more effective in posterior expansion than CBs. However, further high-level studies are warranted to confirm the results [7].

It is generally accepted that the shape and width of the dental arches must be maintained during orthodontic treatment. The characteristic expansion of the arches in SLB appliances (especially Damon®) is linked to a particular arch form, which is the same in both arches, that tends to be expanded in the premolar area, in order to reduce the so-called lateral black corridors when smiling. There have been claims regarding a hypothetical stability in SLB treatments, based upon the theoretical fact that the reduced force needed to obtain orthodontic movement might result in more physiological tooth movements, without overpowering the musculature or obliterating the periodontal vascular vessels [4]. Despite that, clinical and scientific evidence is generally lacking [8, 9].

A retrospective controlled study compared the treatment effects of a passive self-ligating system versus an untreated control sample by using digital dental casts [10]. The passive self-ligating system produces a modest but statistically significant widening of both dental arches. No significant changes in crown torque were detected, but these increases in arch widths are associated with modest significant net gains in maxillary and mandibular arch perimeters (about 2.5 mm) [10].

This was further confirmed on CBCT scans and digital models by Cattaneo et al. [11] which stated that the expansion of the maxillary arch was achieved by buccal tipping of the posterior teeth.

Only a few reports evaluate the long-term effects of SLBs on transverse dimensions of maxillary and mandibular arches [1, 12, 13].

One retrospective study evaluated the long-term effects of SLBs on transverse dimensions of arches and skeletal and soft tissues. The increase in transverse dimensions of the arches remained stable after 2 years from the end of treatment in all 24 subjects analyzed. Again, another most recent retrospective study aimed to analyze any effects on transverse dimension of SLBs in 32 non-extraction cases with a follow-up period of 2 years [2]. After examined dental arches with the use of 3D software, the follow-up analysis showed that transverse expansion did not show any statistically significant relapse, except for slight tendency to restriction in the premolar region [12].

The aim of the present study was to analyze the stability of transverse expansion obtained by SLBs in a larger sample of subjects, in order to evaluate the extent of posterior expansion during active therapy and the rate of relapse in 6-year follow-up period from the end of treatment. Indeed, another objective of the current study was to evaluate whether the majority of relapse movements happened throughout the first-year post-treatment or whether they occurred over a longer period of follow-up.

## Materials and methods

For this retrospective study, a sample was selected, from a pool of patients treated by the same expert operator (WJF), after application of the following inclusion criteria: presence of Class I malocclusion with moderate crowding (3–6 mm or less) and absence of previous orthodontic treatment or permanent tooth extraction. All patients underwent the same archwire sequence and used the same retention protocol. Patients who presented sucking habits, craniofacial syndromes, cysts, cleft lip or palate, and multiple or advanced caries, who needed additional orthodontic anchorage, and patients with incomplete records, were excluded from the study. Patients that had showed a total or partial detachment of the retainer during the follow-up were also excluded from the study. A panoramic radiograph, lateral cephalograms, and dental casts were obtained prior to treatment for all subjects for a proper diagnosis and treatment planning.

All of the patients underwent a non-extractive treatment with Damon®MX self-ligating brackets system (Ormco; Glendora, CA, USA), with standard values of tip and torque and 0.022-in slots. The archwire change sequence was the same for all patients: 0.014 CuNiTi Damon; 0.016 CuNiTi Damon; 0.016 × 0.025 CuNiTi Damon; 0.018 × 0.025 CuNiTi Damon; 0.019 × 025 SS Damon upper and 016 × 025 SS Damon lower.

At the end of treatment lingual fixed retainers from canine to canine were applied in both arches. No removable retainer was prescribed.

After application of the mentioned criteria, the final study sample consisted of 56 Caucasian subjects (33 females and 23 males) with a mean age of 16.9 ± 9.0 years when orthodontic treatment started.

**Fig. 1:**
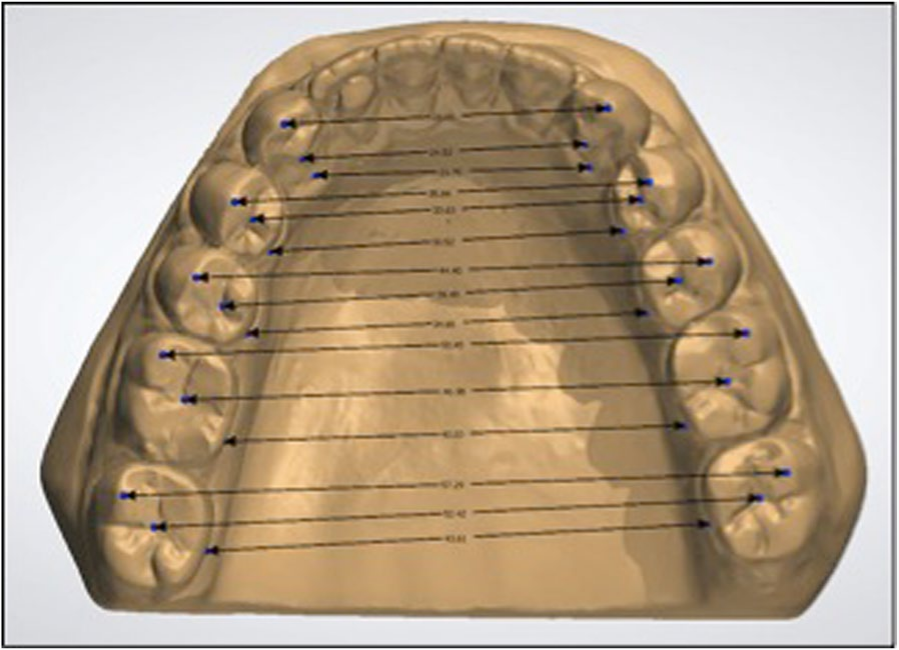
Example of the measure digitization on a lower model

Maxillary and mandibular 3-dimensional (3D) models of each patient were obtained at four timepoints: before treatment (T0), immediately after debonding (T1), 1 year after (T2) and six years after treatment (T3). The models were measured with Orthoanalyzer software (3Shape, Copenhagen, Denmark), and three different transverse linear measures were obtained for each model (Fig. 1):

- The transverse intercusp distances were calculated as the distance from the cusps of the canine, from the vestibular cusps of the bicuspids and from the mesiovestibular cusps of molars.
- The transverse centroid distances were obtained as the measurement of the distance between the midpoint between mesial and distal points and the midpoint between the gingival point of the facial axis of the clinical crown and the gingival point of the lingual side.
- The transverse lingual distances were measured as the distance between the gingival lingual points of analogous teeth.

The transverse diameters were measured for all teeth by the same operator (RF) starting from the canines to the second molars, for a total of 1680 observations. In order to ease the following interpretation of the dataset, the subsequent statistical analysis was performed on the average values ​​obtained from each single pair of homologous elements.

### Statistical analysis

The analysis was performed by one operator (SGA) under the linear mixed regression framework, considering the time as within subject factor and the subject as random factor; the side was inserted in the model as control factor. A post hoc analysis using the emmeans R package allowed to identify which time pairs could be deemed statistically different.

Method error was assessed by repeating 400 randomly selected measures after a 2-week interval by the same operator, and Dahlberg’s D was calculated.

The R Statistical software was used to perform the analyses. Statistical significance was assessed using a type-I error threshold of α = 0.05, while the power threshold set is 1 − β = 0.80. Taking into account the collected sample size, the number of repeated measures and the reference levels for α and β, a lower threshold for the minimum detectable effect size of the study is *f* = 0.158 that lies between a “small” and “medium” reference level.

## Results

Measurement method analysis confirmed that there were no systematic measurement errors (Table 1).

**Table 1:** Method analysis

	T0		T1		T2		T3	
	Ttest_pvalue	Dahlberg	Ttest_pvalue	Dahlberg	Ttest_pvalue	Dahlberg	Ttest_pvalue	Dahlberg
3–3 upper	0.4924	0.0044	0.809	0.021	0.6824	0.025	0.4502	0.059
4–4 upper	0.5632	0.084	0.7034	0.049	0.5835	0.067	0.1140	0.056
5–5 upper	0.1713	0.062	0.6346	0.078	0.7751	0.032	0.1909	0.017
6–6 upper	0.1171	0.124	0.4383	0.057	0.0675	0.034	0.9461	0.042
7–7 upper	0.7995	0.028	0.4069	0.018	0.0091	0.040	1.0000	0.019
3–3 lower	0.2567	0.057	0.1987	0.052	1.0000	0.082	0.0470	0.038
4–4 lower	0.7747	0.034	0.7372	0.076	0.9673	0.039	0.2570	0.057
5–5 lower	0.5125	0.028	0.7822	0.052	0.0760	0.038	0.3361	0.132
6–6 lower	0.5422	0.099	0.3043	0.073	0.1795	0.047	0.4875	0.071
7–7 lower	0.1375	0.063	0.0751	0.169	0.4193	0.108	0.7008	0.075

There were increases in all transverse dental measurements during active treatment, including inter-molar ant inter-canine width (Tables 2, 3).

**Table 2:** Maxillary transverse dimensions (mm) before treatment (T0), immediately after treatment (T1), 1 year after (T2) and 6 years after the end of treatment (T3)

		T0	DS	T1	DS	T2	DS	T3	DS
3–3 upper	Cusp	32.52	2.78	34.97	2.12	35.09	1.81	35.31	1.83
	Centroid	29.00	2.51	30.27	1.81	30.35	1.67	30.53	1.64
	Lingual	24.94	2.60	25.52	1.63	25.59	1.36	25.66	1.37
4–4 upper	Cusp	39.52	2.97	42.78	4.31	42.80	1.92	42.36	2.18
	Centroid	34.22	2.71	37.49	2.34	37.10	1.55	36.56	1.86
	Lingual	27.28	2.87	30.49	2.25	29.71	2.25	29.45	2.20
5–5 upper	Cusp	44.78	3.76	48.18	2.10	47.62	2.11	47.29	2.40
	Centroid	39.42	3.42	42.38	1.88	41.82	1.95	41.45	2.24
	Lingual	32.38	3.59	35.11	2.68	34.55	2.70	34.14	2.64
6–6 upper	Cusp	50.97	3.22	53.24	2.54	52.55	2.90	52.64	2.63
	Centroid	45.61	2.94	47.00	2.23	46.88	2.31	46.73	2.55
	Lingual	36.46	3.87	37.66	3.73	37.22	3.56	37.28	3.42
7–7 upper	Cusp	55.96	3.23	57.85	3.08	57.79	2.90	58.21	3.18
	Centroid	50.36	3.14	51.68	2.97	51.62	2.85	52.12	3.16
	Lingual	40.63	4.09	42.68	4.03	42.33	3.83	42.69	4.05
3–3 lower	Cusp	25.53	2.15	26.89	1.67	26.89	1.51	26.79	1.59
	Centroid	22.97	1.69	23.92	1.35	23.76	1.06	23.74	1.23
	Lingual	20.17	2.02	21.51	1.07	21.34	0.92	21.40	1.10
4–4 lower	Cusp	32.50	3.11	35.05	1.84	34.70	1.61	34.45	2.08
	Centroid	29.46	2.59	31.83	1.49	31.33	1.41	30.85	1.77
	Lingual	26.25	2.67	28.58	1.29	27.89	1.78	27.80	1.83
5–5 lower	Cusp	38.53	3.59	41.20	1.85	40.18	1.91	39.40	2.28
	Centroid	34.76	3.41	37.05	1.55	36.10	1.72	35.34	2.07
	Lingual	30.87	3.00	32.39	2.04	31.86	1.66	31.33	2.09
6–6 lower	Cusp	44.75	3.70	46.57	2.51	45.95	2.14	45.63	2.42
	Centroid	41.03	2.58	42.26	2.00	41.68	1.99	41.35	2.45
	Lingual	35.05	2.63	35.83	2.14	35.48	2.41	35.74	2.72
7–7 lower	Cusp	49.32	4.30	52.99	3.05	52.05	2.80	52.19	3.16
	Centroid	46.18	2.76	47.83	4.53	47.77	2.35	47.76	2.51
	Lingual	40.18	2.78	41.81	2.36	41.20	2.57	41.65	2.62

**Table 3:** Statistical comparison between transverse dimensions (mm) at T0, T1, T2 and T3

	T0-T1				T0-T2				T0-T3				T1-T2				T1-T3				T2-T3			
	Value	*Sig*	LCI	UCI	Value	*Sig*	LCI	UCI	Value	*Sig*	LCI	UCI	Value	*Sig*	LCI	UCI	Value	*Sig*	LCI	UCI	Value	*Sig*	LCI	UCI
3–3 upper	− 1.38	*P* < *0.01*	− 1.76	− 1.00	− 1.47	*P* < *0.01*	− 1.85	− 1.09	− 1.63	*P* < *0.01*	− 2.01	− 1.25	− 0.09	*NS*	− 0.44	0.26	− 0.25	*NS*	− 0.60	0.10	− 0.16	*NS*	− 0.50	0.19
4–4 upper	− 3.25	*P* < *0.01*	− 3.78	− 2.72	− 2.87	*P* < *0.01*	− 3.40	− 2.34	− 2.45	*P* < *0.01*	− 2.98	− 1.92	0.39	*NS*	− 0.15	0.92	0.80	*P* < *0.01*	0.27	1.33	0.41	*NS*	− 0.12	0.94
5–5 upper	− 3.01	*P* < *0.01*	− 3.46	− 2.56	− 2.45	*P* < *0.01*	− 2.90	− 2.00	− 2.08	*P* < *0.01*	− 2.53	− 1.63	0.56	*P* < *0.05*	0.11	1.00	0.93	*P* < *0.01*	0.49	1.37	0.37	*NS*	− 0.07	0.82
6–6 upper	− 1.58	*P* < *0.01*	− 2.08	− 1.08	− 1.16	*P* < *0.01*	− 1.66	− 0.66	− 1.16	*P* < *0.01*	− 1.66	− 0.66	0.41	*NS*	− 0.08	0.91	0.41	*NS*	− 0.08	0.91	0.00	*NS*	− 0.50	0.50
7–7 upper	− 0.73	*P* < *0.05*	− 1.34	− 0.11	− 0.59	*NS*	− 1.20	0.02	− 0.97	*P* < *0.01*	− 1.58	− 0.36	0.14	*NS*	− 0.36	0.64	− 0.24	*NS*	− 0.74	0.26	− 0.38	*NS*	− 0.87	0.12
3–3 lower	− 1.19	*P* < *0.01*	− 1.50	− 0.88	− 1.08	*P* < *0.01*	− 1.39	− 0.77	− 1.04	*P* < *0.01*	− 1.35	− 0.73	0.11	*NS*	− 0.19	0.41	0.14	*NS*	− 0.16	0.45	0.04	*NS*	− 0.27	0.34
4–4 lower	− 2.42	*P* < *0.01*	− 2.85	− 1.99	− 1.90	*P* < *0.01*	− 2.34	− 1.47	− 1.63	*P* < *0.01*	− 2.06	− 1.19	0.52	*P* < *0.05*	0.08	0.95	0.79	*P* < *0.01*	0.35	1.23	0.27	*NS*	− 0.16	0.71
5–5 lower	− 2.19	*P* < *0.01*	− 2.67	− 1.71	− 1.36	*P* < *0.01*	− 1.84	− 0.88	− 0.68	*P* < *0.01*	− 1.16	− 0.19	0.83	*P* < *0.01*	0.36	1.30	1.51	*P* < *0.01*	1.04	1.99	0.68	*P* < *0.01*	0.21	1.16
6–6 lower	− 1.36	*P* < *0.01*	− 1.83	− 0.90	− 0.84	*P* < *0.01*	− 1.31	− 0.38	− 0.62	*P* < *0.01*	− 1.09	− 0.15	0.52	*P* < *0.05*	0.05	0.99	0.74	*P* < *0.01*	0.27	1.21	0.22	*NS*	− 0.25	0.69
7–7 lower	− 1.91	*P* < *0.01*	− 2.65	− 1.18	− 1.41	*P* < *0.01*	− 2.14	− 0.67	− 1.64	*P* < *0.01*	− 2.37	− 0.91	0.51	*NS*	− 0.14	1.15	0.27	*NS*	− 0.37	0.91	− 0.23	*NS*	− 0.87	0.40

Sig, significance; NS, not significant; LCI, lower limit of 95% confidence interval; UCI, upper limit of 95% confidence interval

**Fig. 2:**
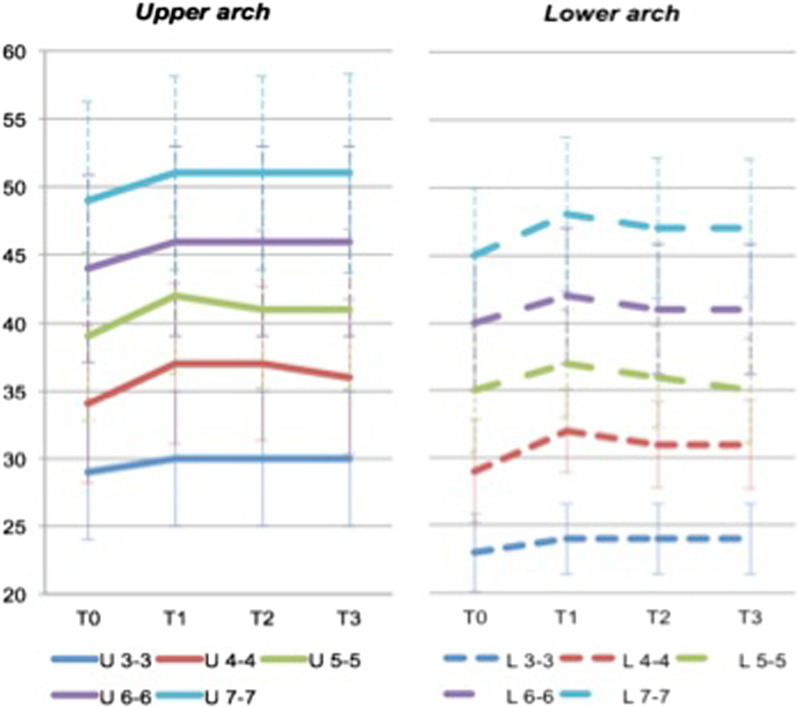
Graphical representation of the mean diameter (*y* axis, in mm) by time (*x* axis), in upper (*left*) and lower arch (*right*). *T0* = *before treatment, T1* = *after treatment, T2* = *1 year after treatment, T3* = *6 year after treatment*

A statistically significant (*p* < 0.05) reduction of the transverse diameter was found, especially for upper and lower premolars, from T1 to T3 (Fig. 2). The most reduction was found lying between T1 and T2 (namely, in the first year after debonding), rather than the following T2-T3 period. The second lower premolar diameter showed the most reduction, reducing from a mean of 37 mm (SD 4.0 mm) to 36 mm (SD 3.8 mm) after one year, to 35 mm (SD 3.9 mm) after six years. A certain increase of the diameter was observed at inter-second molar from T1 to T3; however, this value was found to be not statistically significant and, moreover, not all patients presented the second molars at T0 or, in some cases, they were partially erupted. No relapse was observed at inter-canine, inter-upper first molar and inter-second molar diameters. The presence of lingual fixed retainers should be taken into account when considering the lack of inter-canine relapse.

## Discussion

The stability of orthodontic treatment over time is still today one of the main challenges in orthodontics. Post-treatment assessment of treated malocclusions has been of interest for several decades, and several studies showed that transverse diameters tend to decrease during the post-retention period, especially if they had been expanded during treatment [13]. When possible, maxillary expansion represents the gold standard to correct skeletal transverse deficiency associated with posterior uni- or bilateral crossbite [14]. In growing patients with primary and mixed dentitions, it results in an increased transverse maxillary width and a prevention to impacted canines [15].

In the present study, we evaluated transverse effects of self-ligating appliances on virtual models. The analysis of the dental casts showed that during the active treatment there is an expansion in each sector, mainly at the premolar level in both arches, due to the arch form of the Damon system, which is more expanded at the level of the premolars to prevent black corridors [10]. Other studies [3, 16] found similar results in terms of the capability of STLs to increase dento-alveolar widths during active treatment by buccal tipping of the posterior teeth.

In our study, upper and lower premolars and lower first molars showed a significant reduction in their transverse diameter values one year (T2) and six years after treatment (T3), with respect to the end of treatment (T1). But, when comparing the couple of values at T3 and T2, no statistically different values can be found. This suggests that most of the relapse occurs in the first year post-treatment, and that it reaches a plateau of stability that is maintained up to 6 years post-treatment. This is in partial agreement with the results of a similar study by Lucchese, [12] where they found a tendency to transverse diameter restriction at premolars, even if non-statistically significant, in a 2-year follow-up. These results must be carefully compared, given the different protocols of retentions, which may play a crucial role in determining the amount of relapse.

In this sample, fixed canine-to-canine retainers were used in both arches, and it could explain the lack of transverse relapse at canine level; several studies have shown that fixed retainers could be the right approach to maintain the alignment of the anterior teeth, although there is a lack of high-quality evidence to endorse the use of one type of orthodontic retainer based on risk of failure [17, 18].

One study [2] analyzed a group of 24 patients who had received treatment with Damon3 appliances, assessing the stability of cast measurements and cephalometric values after six months and two years. The conclusion of the study was that, with regard to the cast evaluation, there was a significant relapse in the 2-year follow-up, especially at the upper and lower premolars and upper first molars (second molars were not taken into account). This has been confirmed by the current study which showed similar results regarding the stability of inter-canine diameters, even 6 years after treatment. They also proved a significant relapse in the inter-premolar and inter-molar measures, which was similarly observed in this dataset, with the main difference of upper first molars.

Atik and colleagues [13] aimed to compare the three-year stability out of two different expansion protocols (Damon SLB appliance vs. Quad Helix and Roth prescription-based brackets). All the patients had dentally constricted maxillary arches prior to treatment. Measures were performed on dental casts, measuring the distances between cusps of the same couple of teeth on the same arch. Both groups showed statistically significant increases in all transverse dental measurements during active treatment; in the Damon group, they observed a significant relapse in inter-canine width three years after debonding. It may be important to notice that the retention protocol for all patients contemplated upper and lower removable retainers Hawley type for one year (worn full time for six months and thereafter at night-time for the remaining 6 months). Retention in all the sample was solely based on fixed lingual bonded retainers, which apparently managed to maintain the inter-canine diameters unchanged.

Another paper [^19^] aimed to retrospectively evaluate the stability of various indexes, including inter-canine and inter-molar width, in a SLB group and a conventional brackets group. After a follow-up period of two years and another of 7.24 years, they found that the inter-canine and inter-molar expansion obtained during active treatment tended to stay stable in all the 30 SLB patients. These results also seem to be in agreement with those derived from this study, even if the different retention protocol must be considered (Hawley retainers were used in both arches for approximately 2 years in Yu and colleagues’ study).

While the use of anterior fixed retention from canine to canine is a well-established technique, there are still few indications whether a posterior retention is needed. In the present study, no removable retention (such as essix or Hawley-type retainer) was delivered to patients, so we managed to evaluate the transverse arch expansion and its stability. These results seem to booster that the absence of an adequate retention protocol, especially in the premolar sectors, after the important expansion obtainable with the SLB system, could lead to a relapse within the first year after debonding.

## Conclusions

- All transverse dental measurements showed significant increases during SLB treatment, including inter-molar and inter-canine width.
- The expansion achieved with the therapy has no statistically significant relapse at 6 years from the end of therapy, except for upper and lower premolars.
- Most of the relapse in the upper and lower inter-premolar distance was found in the first year after debonding, rather than in the following follow-up period.
- Inter-canine and inter-molar diameters showed no relapse one and six years after treatment.
- The type of retainer could have an influence in the amount and timing of relapse after SLB treatment.

## Data Availability

The datasets used and/or analyzed during the current study are available from the corresponding author on reasonable request.
