# Correction to: Stability of transverse dental arch dimension with passive self-ligating brackets: a 6-year follow-up study

**DOI:** 10.1186/s40510-022-00428-1

**Published:** 2022-07-27

**Authors:** Franz Josef Willeit, Francesca Cremonini, Paul Willeit, Fabio Ramina, Marta Cappelletti, Giorgio Alfredo Spedicato, Luca Lombardo

**Affiliations:** 1 Brunico, Italy; 2 Postgraduate School of Orthodontics, University of Ferrara, via Luigi Borsari, 46, 44121 Ferrara, Italy; 3 Department of Statistics and Quantitative Methods, University of Milano-Bicocca, Milan, Italy

## Correction to: Progress in Orthodontics (2022) 23:19 https://doi.org/10.1186/s40510-022-00414-7

Following publication of the original article [1], the authors identified an error in the author name Giorgio Alfredo Spedicato. The given names were erroneously transposed.

The incorrect author name is: Alfredo Giorgio Spedicato

The correct author name is: Giorgio Alfredo Spedicato

The author group has been updated above and the original article [1] has been corrected.
